# Voxel-based automatic multi-criteria optimization for intensity modulated radiation therapy

**DOI:** 10.1186/s13014-018-1179-7

**Published:** 2018-12-05

**Authors:** Yanhua Mai, Fantu Kong, Yiwei Yang, Linghong Zhou, Yongbao Li, Ting Song

**Affiliations:** 1 Department of Biomedical Engineering, Southern Medical University, Guangzhou, 510515 Guangdong China; 2 Department of Radiation Oncology, Sun Yat-Sen University Cancer Center,State Key Laboratory of Oncology in South China, Collaborative Innovation Center for Cancer Medicine, Guangzhou, 510060 China; 3 Department of Radiation Oncology, Zhejiang Cancer Hospital, Zhejiang, 310022 Hangzhou China

**Keywords:** Multi-constraints optimization, Voxel-dependent parameter, Treatment planning, Intensity modulated radiation therapy

## Abstract

**Background:**

Automatic multi-criteria optimization is necessary for intensity modulated radiation therapy (IMRT) because of low planning efficiency and large plan quality uncertainty in current clinical practice. Most studies focused on imitating dosimetrists’ planning procedures to automate this process and ignored the fact that organ-based objective functions typically used in commercial treatment planning systems (such as dose-volume function) usually lead to sub-optimal plans. To guarantee the optimum results and to automate this process, we incorporate an improved automation strategy and a voxel-based optimization algorithm to generate a novel automatic multi-criteria optimization framework. We then evaluate it in clinical cases.

**Methods:**

This novel automatic multi-criteria optimization framework incorporates a ranked priority-list based automatic constraints adjustment strategy and an in-house developed voxel-based optimization algorithm. Constraints are sequentially adjusted following a pre-defined priority list. Afterward, a voxel-based fluence map optimization (FMO) with an orientation to the newly updated constraints is launched to find a Pareto optimal solution. Loops of constraints adjustment are repeated until each of them could not be relaxed or tightened. The feasibility of the framework is evaluated with 10 automatic generated gynecology (GYN) cancer IMRT cases by comparing the dosimetric performance with the original.

**Results:**

Plan quality improvement is observed for our automatic multi-criteria optimization method. Comparable DVHs are found for the planning target volume (PTV), but with better organs-at-risk (OAR) dose sparing. Among 13 evaluated dosimetric endpoints, 5 of them show significant improvements in automatically generated plans compared with the original plans. Investigation of improvement tendency during optimization exhibits gradual change as the optimization stage proceeds. An initial voxel-based optimization stage and in-low-priority dosimetric criteria tighten can significantly contribute to the optimization procedure.

**Conclusions:**

We have successfully developed an automatic multi-criteria optimization framework that can dramatically reduce the current trial-and-error patterned planning workload while affording an efficient method to assure high plan quality consistency. This optimization framework is expected to greatly facilitate precise radiation therapy because of its advantages of planning efficiency and plan quality improvement.

## Background

Intensity-modulated radiation therapy (IMRT) treatment plan optimization is a multi-objective problem that aims to provide dose coverage, homogeneity and conformity to planning target volume (PTV) while sparing organs-at-risk (OARs) [1–4]. From a mathematical perspective, directly solving such multi-objective problems can be challenging, whereas a weighted and summed single-objective optimization is more reliable and has been commonly used in commercial treatment planning systems (TPS) [5]. However, the current commercial TPS needs users to input some optimization parameters, including ideal dosimetric goals and the organ-based weighting factors. These factors represent the importance of specific organs that are difficult to assign before optimization. Therefore, the dosimetrists must manually tune these parameters through a trial-and-error process [6–8], which leads to a time-consuming planning procedure; moreover, the final plan’s quality highly depends on dosimetrist’s experience [9–11].

Several studies have attempted to automate multi-criteria plan optimization. The methods that use a pre-defined constraint priority list and a particular constraints adjustment mechanism have been studied for years. Wilkens et al. came up with a four-step enhancement procedure for automatic multi-criteria planning and used this procedure to evaluate head and neck cases [12]. The goal of each step is as follows: 1) to obtain homogeneous dose distributions for PTV; 2) to reduce the mean dose for OARs (i.e., parotid glands and the oral cavity); 3) to reduce doses for other normal tissues; and 4) to smoothen the fluence maps. These procedures are performed sequentially, and the fourth step incorporates a non-clinical goal. Jee et al. [13] implemented a hierarchical method called lexicographic ordering (LO), which was applied to prostate and head and neck cases. The goals for optimization are also pre-categorized into four levels of priority based on clinical importance. During optimization, the objectives were handled individually in a pre-defined order and consequently changed into constraints. Different from these works, Breedveld et al. [14] adopted an automatic constraint adjustment strategy based on a pre-defined priority list to generate a plan with all constraints met as well as possible. The strategy also includes four stages. The first stage tries to find an initial solution. The second stage relaxes the constraints when the initial solution from stage one does not satisfy all constraints. The third and fourth stages attempt to tighten all constraints to their maximum extent without higher-priority constraints. Breedveld et al. [15, 16] later developed an improved automatic multi-criteria plan optimization model based on lexicographic ordering (LO) and showed a close or better plan quality than original plans from the dosimetrists.

However, most previous studies used an organ-based optimization model that has an incomplete mathematical solution space and usually leads to sub-optimal plan quality [17, 18]. Compared with the organ-based optimization model, studies have shown that a refined voxel-based optimization model can help to navigate the solution from the partial space to the entire Pareto surface [18]. In that case, generating an optimal treatment plan with voxel-based optimization model is practical. For example, Cotrutz and Xing [19, 20] first refined the traditional organ-based optimization objective to a voxel-based one and showed that the dose-volume histogram(s) (DVH) could be greatly improved when the dose distribution is on a local level or with differential shapes of the region-of-interest (ROI). Moreover, Wu et al. [21] proposed a novel voxel-based model and mathematically proved that the voxel-based optimization method can carefully balance the trade-offs between ROIs. They also found that re-optimizing original plans by adjusting voxel-based weighting factors is equivalent to tuning voxel-based threshold doses.

Considering that a complete planning optimization procedure is the repeated iterations of optimization under appropriate constraints, we combined an automatic constraints adjustment mechanism with a voxel-based plan optimization algorithm to build a novel automatic multi-criteria optimization framework. This framework automatically adjusted the dosimetric constraints based on their clinical importance and simultaneously explored a wider solution domain with voxel-based optimization model. Constraints are sequentially adjusted based on a pre-defined constraint priority list, and a voxel-based fluence map optimization (FMO) engine with an orientation to the newly updated constraints is then launched to search a Pareto optimal solution. The feasibility of the framework was evaluated with 10 clinically collected gynecology (GYN) cancer IMRT cases by comparing the plans generated by the proposed method with the original clinical ones on plan quality, in terms of DVH curves and their detailed dosimetric endpoints. Furthermore, a paired t-test is performed on detailed dosimetric endpoints to reveal the significant statistical difference between evaluated plans. The transition tendency within the optimization procedures was recorded for further investigation.

## Methods

### Overall framework

**Fig. 1:**
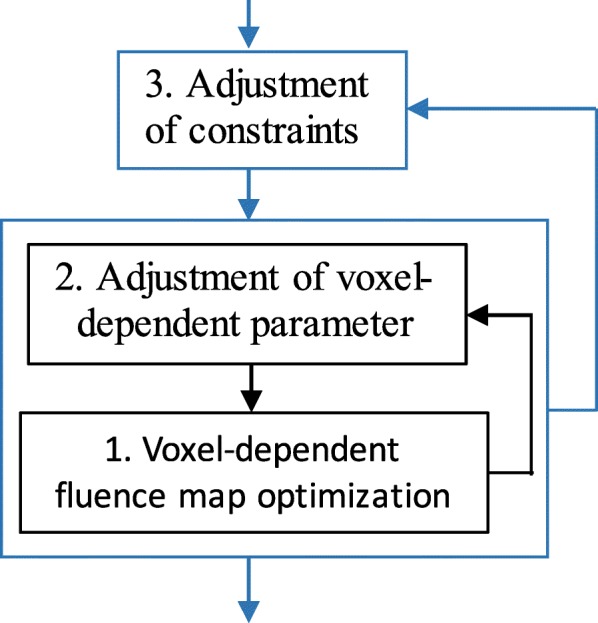
Proposed automatic multi-constraints optimization framework

The proposed automatic multi-criteria optimization framework includes two main loops (Fig. 1), as follows: an outer loop (blue box), to prospect the most appropriate constraints; and an inner loop (black box), to explore the global optimum. The outer loop first adjusts the constraints automatically based on the optimization results from a previous iteration. It relaxes constraints if they are not satisfied, but otherwise tightens them. Whenever the constraints are set, the voxel-based FMO is immediately launched to obtain a Pareto optimal solution under these constraints. The loop is repeated until all constraints cannot be relaxed or tightened further. The inner loop is the mentioned voxel-based FMO. The loop iterates between two parts. One is the automatic adjustment of voxel-based parameters, and the other is the solver of FMO problem for given voxel-based parameters. Loops will stop when all given constraints in the outer loop are met or the iteration reaches its maximum number. Details are stated carefully in the following parts.

### Voxel-based fluence map optimization

Normally, for IMRT optimization, the primary step is to obtain optimal intensity maps for beams that penetrate from assuming known and fixed angles. Here, we implement a voxel-based quadratic model formula below for fluence map optimization [22].

1 $$ {\displaystyle \begin{array}{l}s(f)=\sum \limits_{v\in V}{\xi}_v{\left( Hf-{d}_v^p\right)}^T{\tilde{w}}_v\left( Hf-{d}_v^p\right)+\kappa {(Mf)}^T(Mf)\\ {}\kern7.699995em s.t.\kern0.6em f\ge 0,{D}_{x\%}\ge \left(\le \right){d}_1,{V}_x\le {v}_1\kern1em \end{array}} $$

Notation *f* is the beamlet intensity vector with all nonzero elements. *H* is the dose deposition coefficient matrix pre-calculated using the quadrant infinite beam (QIB) [23, 24] algorithm implemented in a computational environment for radiotherapy research (CERR) platform [25]. The dose distribution *d* is linear to beamlet intensity, and the calculation can be quickly performed by *d=Hf* during optimization.

*s(f)* denotes the objective function of FMO problem, including two terms. The former term is the dose difference between the received dose *Hf* and the reference dose ${d}_v^p$ (prescription dose for target, a reasonable low dose for OAR). The latter term is a regularization term that ensures a smooth fluence map. *κ* is the fluence map smooth weighting factor. *M* is an operator equal to Δ*f*/*f*, Δ*f* is the discrete Laplacian operator to fluence. Two types of voxel related parameters are incorporated in the former part of Eq. (1), namely, the regular organ-based weight *ξ*_*v*_ and the refined voxel-dependent parameter *w*_*j*_, which is the element of diagonal matrix ${\tilde{w}}_v$ with its dimension equals to the number of voxels selected in optimization, *j*. *j* ∈ *v* is the voxel index belongs to ROI *v*. *ξ*_*v*_ is the weight assigned for different ROIs *v* ∈ *V*. *D*_*x*%_ ≥ (≤)*d*_1_ and *V*_*x*_ ≤ *v*_1_ are the commonly used dose-volume constraints. When all parameters in Eq. (1) are set, a bound constrained convex quadratic problems (BOXCQP) algorithm [26] is called to solve the optimization problem, thereby achieving a feasible solution in rapid convergence.

### Adjustment of voxel-based parameters

When sets of constraints were updated in the outer loop of the framework, voxel-based parameters should be tuned consequently to satisfy these constraints. Considering that the voxels involved in optimization are enormous, typically ~ 10^7^, an automatic voxel-based parameter adjustment method should therefore be preferred over traditional manual tuning. The principle of adjusting voxel-based parameter is intuitive. We find the voxel-violated constraints and increase their corresponding values.

### Penalized voxel selection

**Fig. 2:**
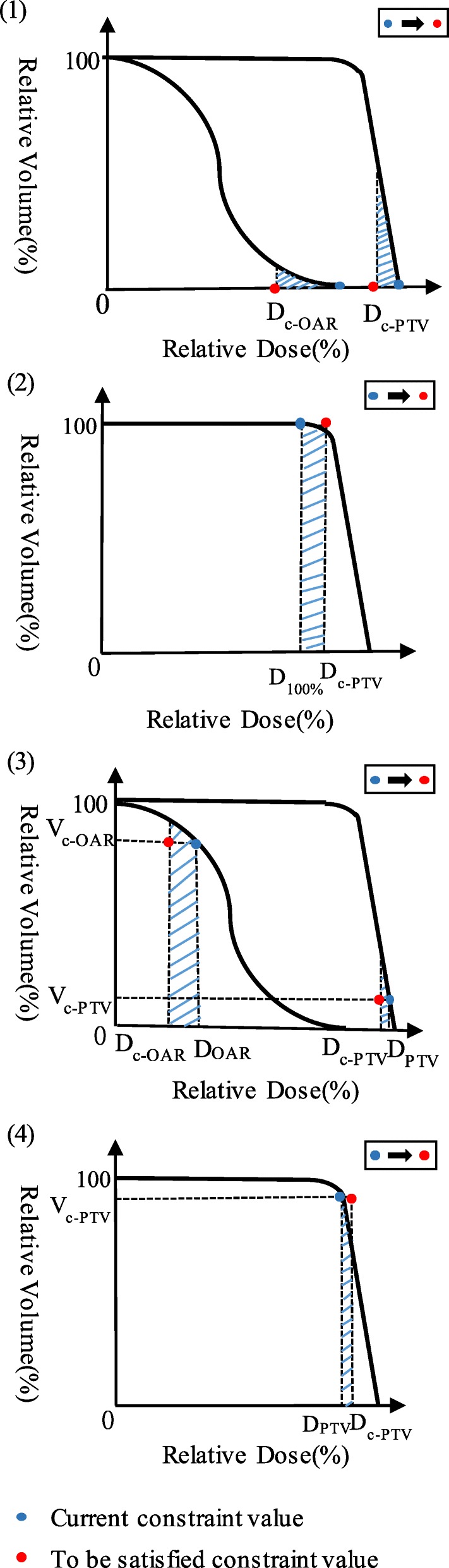
Violation region (shaded area) illustration for PTV and OARs with different constraints. (1) (2) ROIs with constraints of maximum-dose and minimum-dose; (3) (4) ROIs with constraints of maximum and minimum dose-volume

Two main types of constraints are considered in this study, namely, dose and dose-volume constraints. These constraints are commonly used in clinics. Detailed selections for these two situations are shown in Fig. 2. The shaded area is the selected voxel region.

Figure 2 (1) (2) show the selection when constraints are a maximum dose (for PTV and OARs) and minimum dose (for PTV), respectively. *D*_*c*_ is the critical dose level. For maximum dose constraints, voxels with received dose ≥*D*_*c*_ are selected and their assigned parameters are increased. Similarly, for minimum dose constraints, voxels with received dose ≤*D*_*c*_ are selected.

For dose-volume constraints, reducing or increasing doses for voxels with their received dose just over or under the critical dose level is more efficient [27]. Figure 2 (3) (4) show the selection of voxels for maximum dose-volume constraints (for PTV and OARs) and minimum dose-volume constraints (for PTV), *D*_*c*_ is the critical dose level, *V*_*c*_ is the critical volume, *D* is the dose level with a volume equals to *V*_*c*_. For the maximum dose-volume constraint, the voxels with dose value between *D*_*c*_ and *D* (*D* > *D*_*c*_) are selected. For the minimum dose-volume constraint, the voxels with dose values between *D* and *D*_*c*_ (*D* < *D*_*c*_) are selected.

### Voxel-based parameter adjustment

After the selection of penalized voxels, their corresponding parameters are increased accordingly. For minimum dose and dose-volume constraints, voxel-based parameters are updated by ${w}_j^{k+1}={w}_j^k{\left[\left({D}_c+\alpha \right)/{D}_j^k\right]}^{\mu },\left({D}_j^k\le {D}_c\right)$. For maximum dose and dose-volume constraints, voxel-based parameters are updated by${w}_j^{k+1}={w}_j^k{\left[\left({D}_j^k+\alpha \right)/{D}_c\right]}^{\mu },\left({D}_j^k\ge {D}_c\right)$, where ${D}_j^k$ is the received dose for voxel *j* at *k* iteration. ${w}_j^k$ is the voxel-based parameter for voxel *j* at previous iteration *k*. ${w}_j^{k+1}$ is the updated voxel-based parameter for voxel *j* at current iteration *k* + 1. Parameters *μ* and *α*(*α* > 0) are the user-defined enhancement factors. Their effect on the convergence speed are discussed in this paper.

Normally, the optimized dose distribution will meet or become close to the constraints after several iterations of voxel-based parameter update and FMO optimization. When all constraints are met, the iteration is immediately terminated. However, in certain cases, the constraints may be set too tight for this particular patient and are thus hard to meet. Then, the algorithm must force itself to stop by a reasonable maximum iteration time.

### Adjustment of constraints

The promising solution to an efficient and high-quality plan generation is to feed the optimization engine with appropriate constraints based on clinical planning experience. When the given constraints are too tight, the optimization engine cannot easily find a satisfying solution. By contrast, if the constraints are too loose, the achieved plan could be sub-optimal. This situation is the uppermost exact reason for the current trial-and-error planning procedure until the dosimetrists set constraints that are sufficiently tight. Therefore, attempting to automatically adjust these constraints should be addressed (the outer loop in Fig. 1). Prior to the automatic constraints adjustments, a user-defined constraint priority list with some initial values should be given first.

### Constraint priority list

Constraint priority list is a pivotal conversion from the clinical bias to the scientific trade-offs. The list is generated by classifying and sequencing the planning endpoints (normally from clinically adopted protocols) following their clinical importance. Endpoints with their constraints are arranged at different levels, with a corresponding number that indicates its priority. Moreover, constraints are divided into two classes, namely, hard and soft constraints. With a priority number 0, hard constraints cannot be violated and are forbidden to relax or tighten during optimization. By contrast, soft constraints have nonzero priority numbers, and their priorities are decreased gradually with their increasing number. Soft constraints can be relaxed and tightened, and they may be promoted to hard constraints during optimization.

Given an example of priority list set on patients who received IMRT with GYN cancer (Table 1), the optimization requiring dosimetric endpoints and constraints are accordingly derived from a clinical protocol, i.e. the International of Radiotherapy Technology Effectiveness in Cervical Cancer (“INTERTECC”) in this study. Among these, the first imperative criteria would be given to *D*_min_ (the minimum dose), *D*_max_ (the maximum dose), *D*_97%_ (the minimum percentage dose of 97%) of the PTV, and *D*_max_ of the Body-(PTV + 1) (1 cm) for considering the PTV coverage and dose homogeneity and basic normal tissue protection. Thus, they are set as hard constraints with the “highest” priority number of 0. Here “Body-(PTV + 1)” is considered rather than “Body-PTV,” because a dose transition region between high-dose PTV and low dose normal tissue is usually needed to make the optimization problem easier. Other planning-related OAR endpoints are subsequently considered soft constraints, such as endpoints of rectum, bladder, bowel, femoral heads, and bone. Their priorities are co-determined based on both planning experience and physician’s preference. Details are listed as in Table 1.

**Table 1:** Initial constraint priority list for case 1

ROIs	Endpoints	Initial constraints	Priorities
PTV	D_min_	41 Gy	0
PTV	D_max_	50 Gy	0
PTV	D_97%_	43.8 Gy	0
Body	D_max_	50 Gy	0
Body-(PTV + 1)	D_max_	43 Gy	0
Rectum	V_40_	80%	1
Bladder	V_40_	65%	1
Rectum	V_30_	97%	2
Bowel	V_40_	30%	2
Femoral heads	V_30_	15%	2
Bone	V_10_	80%	3
Bone	V_20_	66%	3

### Adjustment strategy

The adjustment of constraints is a four-stage process that has been discussed thoroughly in Breedveld et al. [27]. The first is the preparatory stage. In this stage, we generate an initial dose distribution by applying an in-house developed voxel-dependent parameter optimization. The initial organ-dependent parameters, voxel-dependent parameter (all start from 1), and priority list are the preliminary inputs and are set empirically. Similar to clinical circumstances, constraints set in this stage are consistently too tight. Thus, a solution that can satisfy all the constraints cannot easily be obtained. In this case, partial constraints must be relaxed. This situation leads to the second stage, with an attempt to relax the constraints until each of them can be satisfied. The relaxation begins from low priority to high priority, cluster by cluster. For example, if one or more soft constraints are violated, the lowest priority ones are simultaneously selected for relaxation. A simple but effective means for relaxation is to increase current constraint values with an appropriate interval, such as 0.5% for most cases based on experience. Results generated in this stage have a high possibility to improve. Thus, in the following third and fourth stages, the potential loose constraints should be tightened until the improvement of any single constraint is accompanied by at least one other constraint violation. By contrast, in the tightening round, constraints are adjusted from high-priority to low-priority, endpoint by endpoint. Each stage calls the automated voxel-dependent FMO with a maximum number of iterations. The soft constraints relaxed in the second stage are tightened in the third stage, and the remaining soft constraints are tightened in the fourth stage. If all constraints are met, including the tightened constraints, the optimization system would continue to tighten the remaining ones until it reaches the maximum iteration. Otherwise, the ongoing constraint is reset to its previous value and becomes promoted as a hard constraint. Similarly, a constraint is tightened by subtracting an appropriate interval such as 0.5%.

### Evaluation

The feasibility and efficiency of this proposed automatic multi-criteria optimization framework are evaluated by 10 GYN IMRT cases, with a prescription dose of 45 Gy to the PTV and treated with seven beams (150°, 100°, 50°, 0°, 310°, 260°, and 210°). All these IMRT plans are originally exported from the commercial Eclipse TPS (Varian Medical Systems, Palo Alto, CA). We re-optimized each IMRT plan using this in-house developed automatic multi-criteria optimization framework (called an optimized plan below) and compared it with the original one (called the original plan) in terms of DVH curves and particular endpoints. Beam set-ups are maintained unchanged.

To investigate synoptically the plan quality improvement, a paired t-test was performed on each plan dosimetric endpoint for all evaluated cases between the optimized plan and the original plan. Furthermore, we recorded the stage-status to observe the transition tendency along with the four-stage optimization proceeded by examining their constraint-settings and subsequent optimized values for each dosimetric endpoint.

**Fig. 3:**
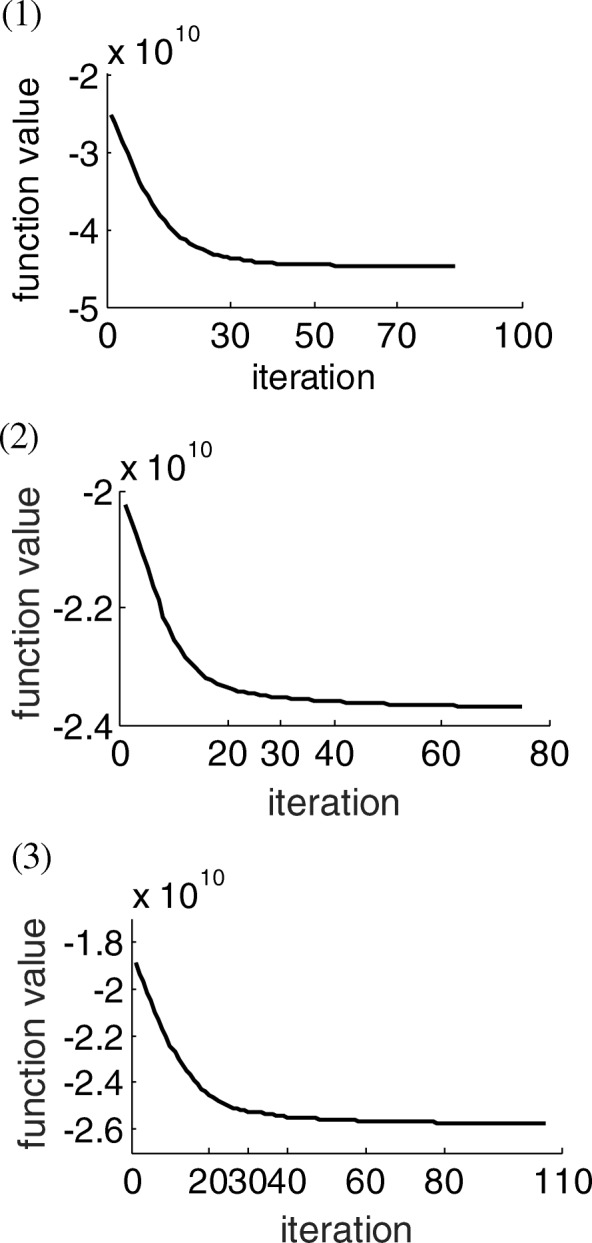
Convergence speed as with voxel-dependent FMO iterations for three cases

**Fig. 4:**
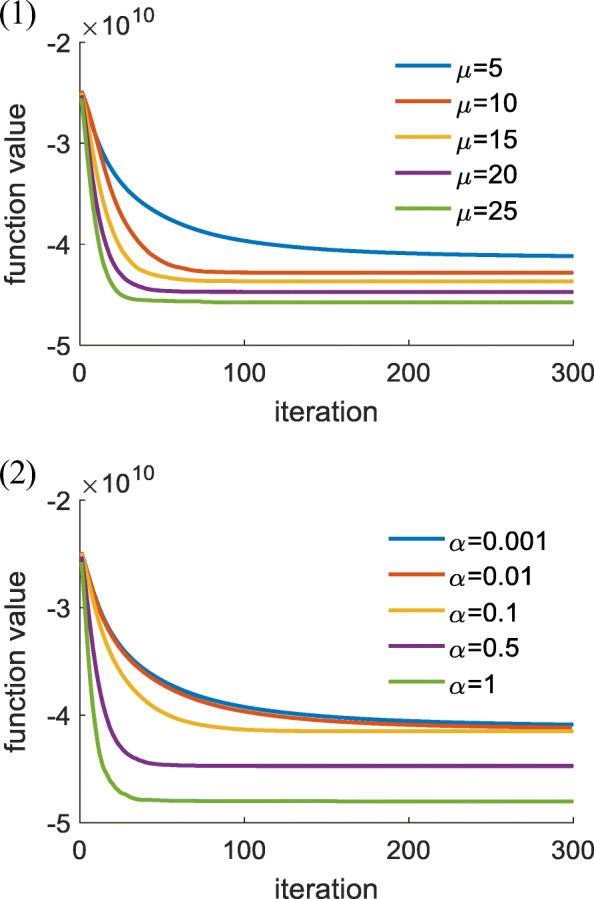
Convergence speed as with various combination of *μ* and *α*. (1): *α* = 0.5, *μ* = 5, 10, 15, 20, 25; (2):*μ* = 20, *α* = 0.001, 0.01, 0.1, 0.5, 1

Other parameters were set by experience, as follows: organ-based weights 100, 1, 2, 0.1, 0.1, 0.1, 0.1 and 0.1 for PTV, bladder, rectum, bowel, femoral heads, bone, Body-(PTV + 1), and body, respectively. The initial voxel-based weight was set to 1, and the initial constraint priority list is shown in Table 1. The reference dose ${d}_v^p$ in Eq. (1) was set to a prescription dose for PTV and empirically for each OAR. Maximum voxel-based parameter updating time was set to 30, 10, 30, and 30 for each stage. Here, 30 is determined based on the convergence of the optimization cost function value as with voxel-weight updating time (Fig. 3). Voxel-dependent parameter auto-tuning influence factors *μ* and *α* were 20 and 0.5, based on the investigation to the convergence when *μ* and *α* ranged from 5 to 25 and 0.001–1, respectively, based on experience (as shown in Fig. 4). Several of these cases couldn’t generate a solution when *μ* was during 20 to 25 owing to an ill-condition Hessian matrix. The same situation occurred when *μ* was equal to 20 and when *α* changed from 0.5 to 1. This whole framework was implemented with Matlab 2013a and installed on a 3.4 GHz Intel Core4 computer running Windows 7.

## Results

### Plan quality improvement

**Fig. 5:**
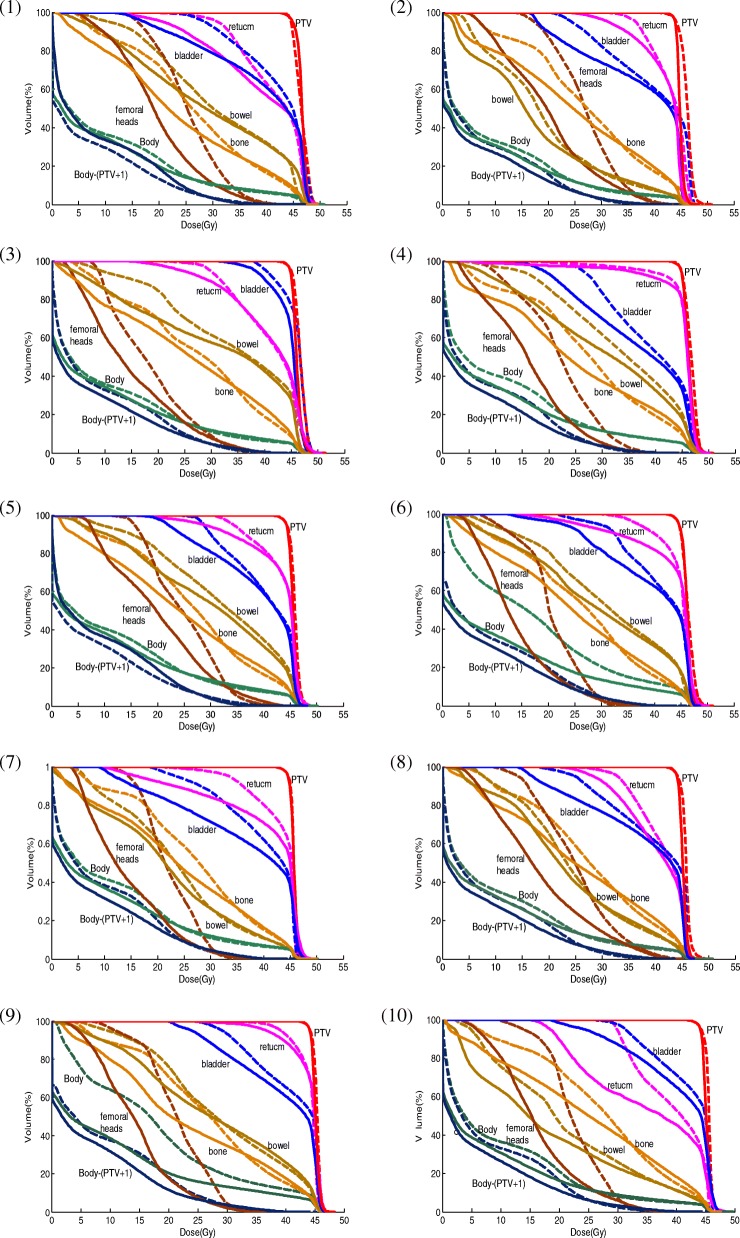
DVH comparisons of the optimized plan and the original for 10 GYN IMRT cases. Solid line: the optimized plan; Dashed line: the original plan

Both DVH curves and particular endpoint comparisons show plan quality improvement for our optimized plans. The DVH comparison of the optimized plans and the original plans for the evaluated 10 GYN IMRT cases is shown in Fig. 5. The optimized plans not only improved the PTV dose homogeneity (with decreased PTV hotspot) but also provided a better OAR dose sparing for most cases. By contrast, Table 2 further illustrates specific DVH endpoint comparison for case 2 as an example. Almost all the endpoints in both plans satisfy the protocol well, except for one soft constraint, i.e., V_30_ of the rectum, which was violated in both plans because of the large overlapping area with PTV. The other two soft constraints, namely, V_10_ and V_20_ of bone, also failed in the original plan but were satisfied in the optimized one. For PTV, comparable D_97%_ and its cold spot *D*_97_are also maintained in the optimized plan, but hot spots decrease, thereby indicating improved PTV coverage and dose homogeneity. Moreover, for OARs, most endpoints lower in the optimized process.

**Table 2:** Dosimetric endpoint comparison for case 2

ROI	Endpoints	Original plan	Optimal plan	Hard constraints
PTV	D_99%_	42.55 Gy	42.96 Gy	40.5 Gy
PTV	D_97%_	43.7 Gy	43.7 Gy	43.65 Gy
PTV	V_115%_	0	0	1
PTV	V_110%_	0.22	0	10
Rectum	D_max_	47.7 Gy	47.7 Gy	51.75 Gy
Bladder	D_max_	48.1 Gy	46.5 Gy	51.75 Gy
Bowel	D_max_	49.3 Gy	47.3 Gy	51.75 Gy
Femoral Heads	D_max_	44.9 Gy	43.5 Gy	51.75 Gy
Bone	V_10_	88.72%	79.65%	90%
Bone	V_20_	74.16%	61.24%	75%
				Soft constraints
Rectum	V_30_	99.97%	96.40%	60%
	V_45_	39.71%	12.01%	50%
Bladder	V_45_	44.64%	13.13%	50%
Bowel	V_40_	7.44%	8.11%	30%
Femoral Heads	V_30_	30.02%	13.87%	15%
Bone	V_10_	88.72%	79.65%	80%
Bone	V_20_	74.16%	61.24%	66%

**Fig. 6:**
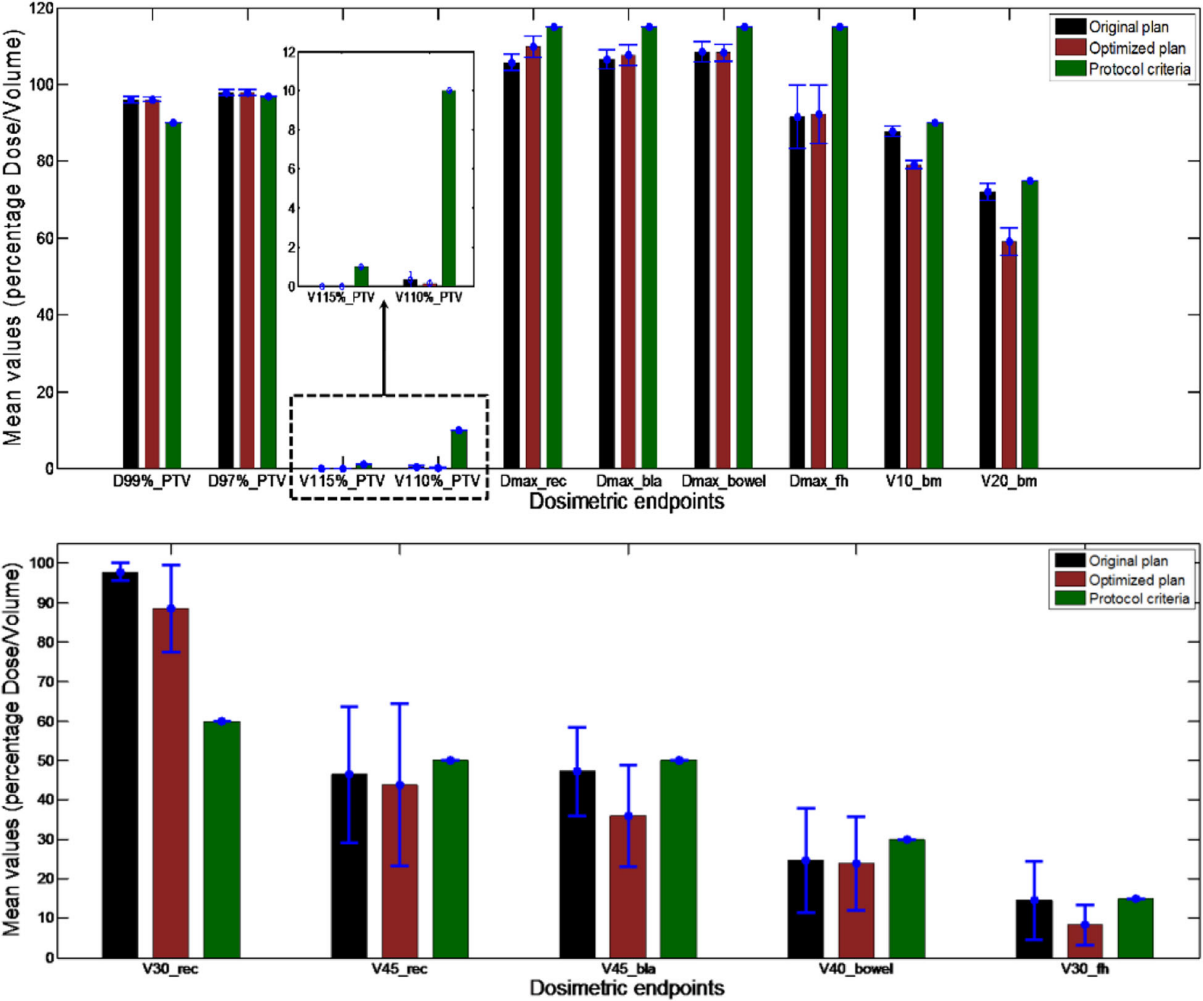
The general comparison of dosimetric endpoints for ten GYN IMRT cases. (1) Hard constraints; (2) Soft constraints

Averaged plan dosimetric endpoints for all evaluated 10 GYN IMRT cases are also investigated, as shown in Fig. 6. For PTV, the D_97%_ and V_115%_ are maintained, whereas the average D_99%_ changes from 43.26Gy (±0.41Gy) to 43.24Gy (±0.26Gy), and the V_110%_ decreased from 0.15% (±0.18%) to 0.06% (±0.10%), for the original and optimized plans, respectively. The average maximum dose of bowel, V_45_ of rectum and bladder, V_30_ of rectum and femoral heads, V_20_ and V_10_ of bone are decreased from 48.83Gy (±1.21Gy) to 48.72 Gy (±1.00Gy), 46.38% (±0.17%) to 43.81% (±0.21%), 47.23% (±0.11%) to 35.96% (±0.13%), 97.73% (±0.02%) to 88.47% (±0.11%), 14.53% (±0.10%) to 8.27% (±0.05%), 72.05% (±0.02%) to 59.02% (±0.04%), and 87.85% (±0.01%) to 79.18% (±0.01%), for the original and optimized plans, respectively. With slightly different trade-offs, the average maximum dose of rectum, bladder and femoral heads increase from 47.57Gy (±0.96 Gy) to 49.4 Gy (±1.29 Gy), 47.95Gy (±1.11 Gy) to 48.42Gy (±1.18 Gy), and 41.24 Gy (±3.73 Gy) to 41.52 Gy (±3.42 Gy), respectively. However, these values effectively satisfy the corresponding constraints.

Results on difference significance analysis between the optimized plan and the original plan on all 10 cases are listed in Table 3. Among 13 observed endpoints, 6 are shown with significant differences between the original and optimized plans (*P* < 0.05), such as D_max_rec_, V_30_rec_, V_45_bla_, V_10_bm_, V_20_bm_, and V_30_fh_.

**Table 3:** Paired-T test of dosimetric endpoints

ROI	Endpoint	*P* value
PTV	D_99%_	0.851042
PTV	V_110%_	0.184812
Bladder	D_max_	0.234172
Bowel	D_max_	0.787316
Femoral heads	D_max_	0.563803
Bowel	V_40_	0.283576
Rectum	V_45_	0.496151
Rectum	D_max_	**0.000459**
Rectum	V_30_	**0.010815**
Bladder	V_45_	**0.004416**
Bone	V_10_	**1.08E-08**
Bone	V_20_	**3.63E-06**
Femoral heads	V_30_	**0.005641**

Bold values indicate significant differences between the original and optimized plans (*P* < 0.05)

### Improvement tendency within optimization

As mentioned above, four stages were designed in this multi-criteria optimization framework, namely, the first preparatory initial stage, the second violated constraint relaxing stage, the third backward relaxed constraint tightening stage, and the last constraint further tightening stage. Plan quality was inspected in the entire time with progression of the stages, and constraints changed during optimization.

**Fig. 7:**
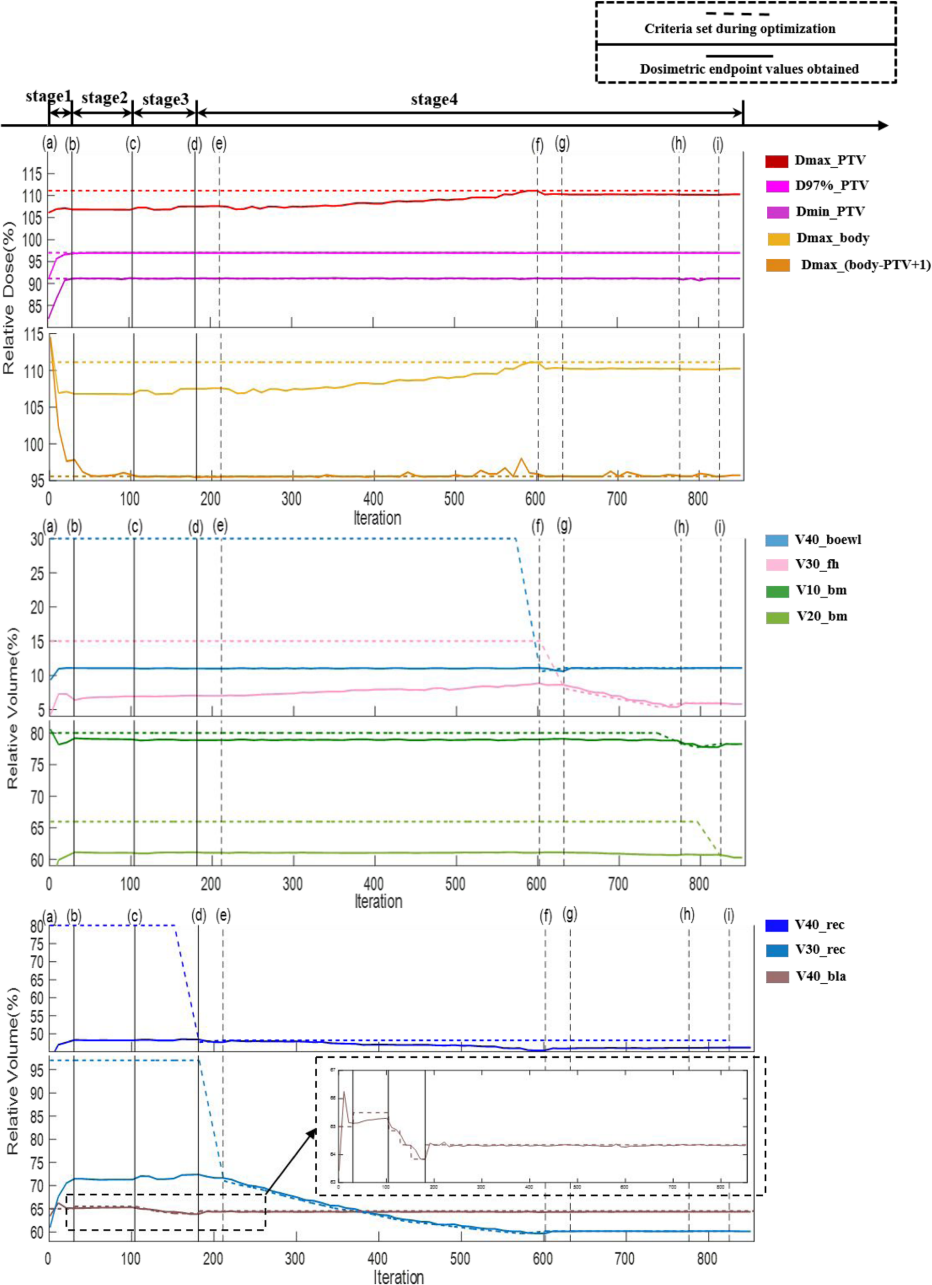
Changing process of clinical relevant constraint and corresponding dosimetric endpoint value for cases 10. Dashed line: clinical relevant constraint; Solid line: corresponding dosimetric endpoint

Figure 7 shows an example of the clinical-relevant dostrimetric endpoint value changing process, with respect to its corresponding pre-defined constraints. The first stage ((a)–(b)) contains 30 voxel tuning iterations and no constraint adjustment. Normally, at the end of this stage, we cannot easily find a plan that meets all constraints, thereby resulting in the second stage, namely, relaxing violated constraints. This second stage begins at the violation of V_40_bla_ and relaxing it from 65 to 65.5%and ends at the plan V_40_bla_ was increased from 65.12 to 65.36% after optimization, requiring another 74 iterations for this case ((b)–(c))).In the third stage, we attempted to undo the constraint relaxations of stage 2 begins at 104th iterations. Between (c) and (d), the algorithm minimizes the dose-volume constraints for the bladder, because the dose-volume constraint for the bladder is violated and relaxed previously. After optimization, the plan V_40_bla_ was decreased from 65.36 to 64.34% at the end of stage 3. At stage 4, first, the V_40_rec_ constraint is tightened, and until the algorithm failed to find a solution ((d)–(e)). Then, the V_30_rec_ is considered. It undergoes a considerable reduction ((e)–(f)). Finally, the V_40_bowel_ ((f)–(g)), V_30_f_ ((g)–(h)), V_10_bm_ ((h)–(i)) andV_20_bm_ ((i)–(j)) are tightened in turn. The entire optimization requires 855 iterations of voxel-based FMO optimization, which requires approximately 30 min to complete.

**Fig. 8:**
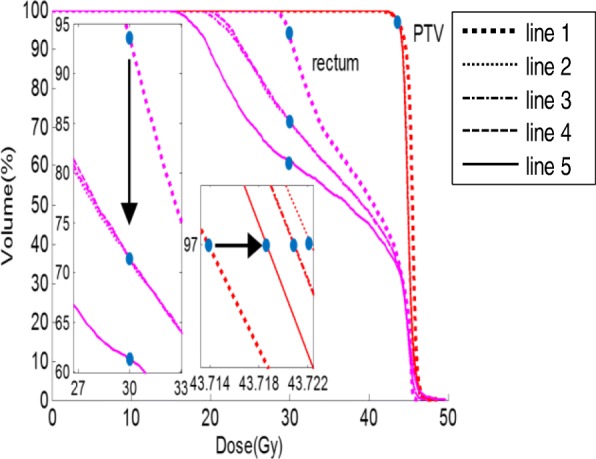
Record on DVH change with each stage ends for case 10. Line 1: Original plan; line 2: Stage 1; line 3: Stage 2; line 4: Stage 3; line 5: Stage 4

Table 4 and Fig. 8 illustrate an example of specific dosimetric endpoint values and its corresponding DVH curves at the end of each stage. Both of the dosimetric endpoint values and their corresponding DVH curves gradually change as optimization proceeds. Relative drastic variations normally occur at the end of stages 1 and 4; for example, the V_30_ of rectum decreases to 71.31 and 61.30%, respectively, compared with the original (93.51%).

**Table 4:** Record on dosimetric endpoint value change with each stage ends for case 10

ROI	Endpoint	Original plan	Stage 1	Stage 2	Stage 3	Stage 4
PTV	D_99%_	42.76 Gy	42.92 Gy	42.89 Gy	42.91 Gy	42.86 Gy
PTV	D_97%_	43.7 Gy	43.7 Gy	43.7 Gy	43.7 Gy	43.7 Gy
PTV	V_115%_	0	0	0	0	0
PTV	V_110%_	0	0	0	0	0
Rectum	D_max_	46.1 Gy	47.3 Gy	47.5 Gy	47.5 Gy	49.7 Gy
Bladder	D_max_	46.5 Gy	47.5 Gy	47.7 Gy	47.9 Gy	48.1 Gy
Bowel	D_max_	46.5 Gy	47.5 Gy	47.7 Gy	47.9 Gy	47.7 Gy
Femoral heads	D_max_	36.5 Gy	38.9 Gy	39.7 Gy	39.9 Gy	38.3 Gy
Rectum	V_45_	21.5%	16.23%	15.54%	15.71%	17.43%
Bladder	V_45_	45.78%	30.07%	29.47%	30.01%	32.28%
Rectum	V_30_	93.51%	71.31%	71.43%	71.41%	61.30%
Bowel	V_40_	11.25%	11.07%	10.88%	10.95%	10.98%
Femoral heads	V_30_	6.71%	6.34%	6.88%	6.92%	5.75%
Bone	V_20_	73.80%	61.1%	60.82%	60.99%	60.66%
Bone	V_10_	88.80%	79.09%	78.71%	78.81%	78.12%

## Discussion

For radiation therapy multi-criteria optimization, appropriate dosimetric goal setting and tuning methods are important. By considering these factors, we have successfully developed an automatic multi-criteria optimization framework with an automatic constraint adjustment strategy and a constraint-oriented voxel-based FMO method. The automatic multi-criteria optimization mechanism is similar to Breedveld’s, but some changes were made, as follows. 1) Initial constraints are determined by experience rather than direct extraction from protocols, thereby providing an improved starting point for the optimization. 2) A more aggressive voxel tuning regime is used to accelerate the updating of the voxel weighting factor based on the difference from calculated dosimetric endpoint value to its criteria.

Ten clinical GYN cancer IMRT cases are used to evaluate the proposed system. The optimized plan not only improves the PTV dose homogeneity (with decreased PTV hotspots) but also provides a better OAR dose sparing for most cases. Among all the investigated dosimetric endpoints, most of them show significant improvement for the optimized plan (*P* < 0.05). Detailed improvement tendency within the optimization procedure is also studied, and every stage is imperative. The plan quality changes most in stages 1 and 4. Although the D_max_ of rectum increases significantly, it still satisfied the protocol defined constraints, possibly because the D_max_ of the rectum is set as a hard constraint and is thus not allowed to be adjusted during the entire optimization. Considering that the adjustment of the constraints and subsequent voxel-dependent parameter-based FMO are fully automatic, the proposed automatic multi-criteria optimization framework can dramatically decrease the current trial-and-error planning workload, thereby affording an efficient means to assure high plan quality consistency.

As depicted by the framework, a physician’s clinical preference is paraphrased by the pre-defined constraint priority list. Thus, the final results embody their expected trade-offs. Consequently, the optimization result should be affected by the initial constraint value and their priorities. Our study trials show that constraints’ ranking, especially for hard constraints, could dramatically affect the optimization results; even only one property change (i.e., priority) can influence optimization. For example, when the priority of the Dmax in body changes from priority 0 to 1, the dose conformity changes dramatically and is far from the clinically accepted plan quality. For those ranked in soft constraints, by initial constraint value changes, the optimal DVH remains in shape. These factors could indicate the robustness of the proposed automatic multi-criteria optimization.

Furthermore, because the mathematical relationship between adjusting voxel-dependent parameters and satisfying DVH constraints remains unknown, the method we use to adjust voxel-dependent parameter is intuitive. Although physical dose sparing can be observed for the proposed optimized plan, the clinical benefit and the efficiency to plan generation in additional tumor sites should be further investigated and evaluated.

## Conclusions

We have successfully developed an automatic multi-criteria optimization framework with an automatic constraint adjustment paradigm and a constraint-oriented voxel-based FMO method. The framework can dramatically reduce the current trial-and-error planning workload and afford an efficient method to assure high plan quality consistency.

## Abbreviations

BOXCQP: Bound constrained convex quadratic problems
CERR: Computational environment for radiotherapy research
DVC: Dose-volume-constraint
DVH: Dose-volume histogram
FMO: Fluence map optimization
GYN: Gynecology
IMRT: Intensity modulated radiation therapy
INTERTECC: International of Radiotherapy Technology Effectiveness in Cervical Cancer
LO: Lexicographic ordering
OAR: Organs-at-risk
PTV: Planning target volume
QIB: Quadrant infinite beam
ROI: Region-of-interest
TPS: Treatment planning system
